# A simple score for the prediction of stent thrombosis in patients with ST elevation myocardial infarction: TIMI risk index

**DOI:** 10.15171/jcvtr.2019.31

**Published:** 2019-08-07

**Authors:** Tufan Çınar, Yavuz Karabağ, Cengiz Burak, Veysel Ozan Tanık, Mahmut Yesin, Metin Çağdaş, İbrahim Rencüzoğulları

**Affiliations:** ^1^Health Sciences University, Sultan Abdülhamid Han Training and Research Hospital, Department of Cardiology, Istanbul, Turkey; ^2^Kafkas University Faculty of Medicine, Department of Cardiology, Kars, Turkey; ^3^Ankara Dışkapı Yıldırım Beyazıt Training and Research Hospital, Department of Cardiology, Ankara, Turkey

**Keywords:** TIMI Risk Index, ST Elevation Myocardial Infarction, Stent Thrombosis, Primary Percutaneous Coronary Intervention

## Abstract

***Introduction:*** The present study aimed to evaluate the potential utility of thrombosis in myocardial infarction (TIMI) risk index (TRI) for the prediction of stent thrombosis (ST) in ST elevation myocardial infarction (STEMI) patients who were treated with primary percutaneous coronary intervention ( pPCI ).

***Methods:*** This retrospective study was related to the clinical data of 1275 consecutive STEMI patients who underwent pPCI from January 2013 to January 2018. The TRI was calculated for each patient, and the following equation was used; TRI = heart rate x [age/10]2/systolic blood pressure. For the definition of ST, the criteria as proposed by the Academic Research Consortium were applied.

***Results:*** The incidence of ST was 3.2% (n=42 patients) in the study. The median value of the TRI was significantly elevated in patients with ST compared to those without ST (22 [17-32] vs. 16 [11-21], *P*<0.001, respectively). In a multivariate logistic regression analysis, the TRI was an independent predictor of ST (odds ratio [OR]: 1.061; 95% CI: 1.038-1.085; *P*<0.001). In a receiver operating characteristic curve analysis, the optimal value of the TRI for the prediction of ST was 25.8 with a sensitivity of 45.2% and a specificity of 86.4%.

***Conclusion:*** The present study finding has demonstrated that the TRI may be an independent predictor of ST in STEMI patients who were treated with pPCI . To the best of our knowledge, this is the first study in the literature in which the TRI and its relationship with ST was evaluated in STEMI patients treated with pPCI .

## Introduction

According to current guidelines, primary percutaneous coronary intervention (pPCI), where available, is the best treatment modality in patients who present with ST elevation myocardial infarction (STEMI).^1^ However, even though an successful restoration of coronary blood flow after a pPCI, some patients during in-hospital stay may develop stent thrombosis (ST), which is the most fearful complication of pPCI. Prior studies demonstrated that the development of ST was associated with elevated mortality rates, especially in patients after pPCI.^2,3^ Despite the improvements in PCI techniques and stent technology, patient’s hemodynamic instability, namely elevated heart rate and low systolic blood pressure, may also play a significant role in the development of ST.^4,5^ In addition, older age is shown to be an independent predictor of the development of ST.^6^ Thus far, some risk scores have been investigated to determine the risk of ST in patients with STEMI.^7,8^ However, most of these scores depend on the complex calculation and include some laboratory parameters that are unknown at the time of pPCI. Therefore, clinicians need a simple and readily available bedside risk score in a clinical practice to determine this dreadful complication of pPCI.

Thrombosis in myocardial infarction (TIMI) risk index (TRI) is a simple and easy risk score that has been shown to be useful for the prediction of short-and long-term mortality in patients who present with STEMI.^9-11^ The TRI includes non-laboratory depending parameters -such as age, systolic blood pressure, and heart rate- all of which can be easily obtained at the first medical contact. As previously mentioned, some components of the TRI were shown to be related with the risk of ST in patient with STEMI. On the other hand, the suitability of TRI to predict the risk of ST has not been yet explored. Hence, we aimed to evaluate the potential utility of TRI for the prediction of ST in STEMI patients who underwent pPCI.

## Materials and Methods

### Data collection

**Figure 1:**
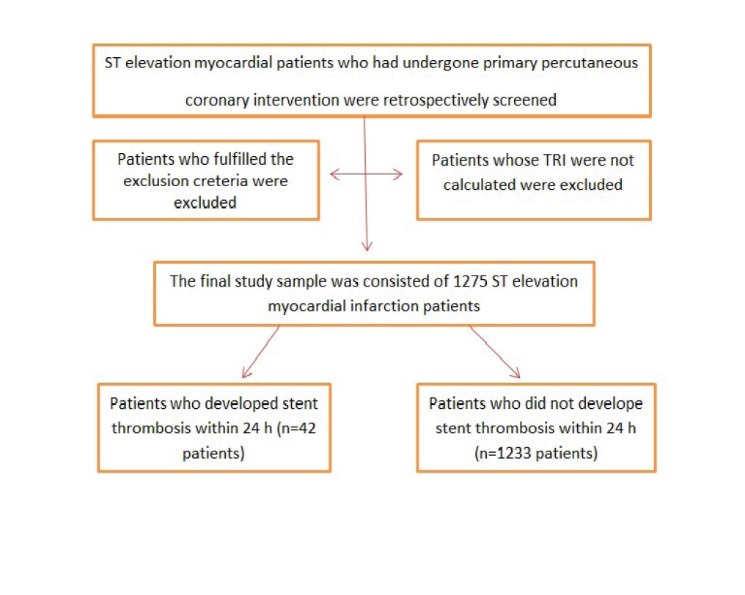
Flow chart of the study population.

This retrospective study was related to the clinical data of 1275 consecutive STEMI patients who underwent pPCI from January 2013 to January 2018. In the present study, patients who had a one or more following criteria were excluded. These criteria were; having acute or chronic infection or acute liver failure, presented with symptoms onset of more than 12 h, underwent thrombolytic therapy or emergent aortocoronary bypass operation due to a failed pPCI. Also, patients with missing clinical data were not included in the study (Figure 1).

In all patients, baseline demographic characteristics and related laboratory and angiographic findings were retrieved from the hospital’s electronic database. In accordance with the current guidelines, all patients received the standard therapy during in-hospital stay.

The TRI was calculated for each patient, and the following equation was used; TRI = heart rate x [age/10]^2^/systolic blood pressure.^9^ The components of the TRI that were used for the calculation including heart rate and systolic blood pressure were obtained at the first medical contact before pPCI. For the definition of ST, the criteria as proposed by the Academic Research Consortium were applied.^12^ In short; the patients who developed ST within 24 h were defined as an acute ST and included in the study. In all patients, two expert interventionist cardiologists who were blinded to all clinical data of the patient confirmed the definitive ST by evaluating the coronary angiographic views.

### Laboratory analysis and echocardiographic examination

All blood samples were obtained from the antecubital vein upon admission to the emergency department. A Beckman Coulter LH 780 device (Beckman Coulter Ireland Inc. Mervue, Galway, Ireland) was used in order to measure all biochemical parameters. Serum cardiac biomarkers, including creatinine kinase-myocardial band and troponin I levels, were obtained upon admission, and their peak values with a 6 h interval were obtained for the final analysis. Modification of Diet in Renal Diseases study equation was used in order to calculate an estimated glomerular filtration rate (eGFR).

In our study, an echocardiographic examination was performed with using a Vivid 7 system (GE Vingmed Ultrasound AS, Horten, Norway) within 24 hours after pPCI. The modified Simpson method was used for the calculation of the left ventricular ejection fraction.

### Coronary angiography and PCI

All coronary angiographic examinations were performed with using the standard catheters via femoral or radial approach. Before the procedure, all patients received 300 mg acetylsalicylic acid along with a loading dose of 300 mg-600 mg clopidogrel. Intravenous unfractionated heparin and additional doses if needed were given in order to achieve an activating clotting time of >250 ms during the procedure. In all patients, a drug-eluting stent(s) was implanted in the infarct- related artery. The choice of an infusion of glycoprotein inhibitors IIb/IIIa, and use of the choice of interventional equipment, including a non-compliant balloon for the postdilatation, was left to the operator’s discretion choice. Before and after the pPCI, all coronary blood flow patterns were subjected to a thorough evaluation for TIMI flow grade using grades I, II, and III.^13^ If the TIMI flow was <3 or the patient had TIMI flow of III with MBG<II, it was defined as angiographic no-reflow. Thrombus burden was assessed according to the TIMI thrombus grading scale that ranged from grade I (no thrombus) to grade V (very large thrombus causing a vessel occlusion).^13^ After the recanalization with a guide-wire or a small balloon, patients with a grade 5 thrombus were reclassified into grade I, II, III, or IV depending on the findings. Using the online syntax score calculator, version 2.1, each lesion with ≥1.5 mm in diameter, and ≥50% stenosis was scored.^14^

### Statistical analysis

IBM SPSS Statistics for Windows, Version 19.0 (IBM Corp. Armonk, NY) was used to carry out the statistical analyses. A mean ± standard deviation was used to express the continuous variables, while frequencies and percentages were used to express the categorical variables. For the comparison of the categorical data, the chi-square or Fisher exact test were performed. The Kolmogorov-Smirnov test was used to test the normality distribution of continuous variables. Multicollinearity between TRI and its components was assessed by the Eigen-value and condition index. Linearity was tested by interacting with the logarithmic transformation of each parameter itself. The Hosmer and Lemeshow statistic of the logistic model was 0.395 (χ^2^=8.40). All relevant variables shown in Tables 1 and 2 were included in a univariate analysis. The independent predictors of ST were identified after performing a multivariate logistic regression analysis. In order to determine the optimal value of the TRI in predicting ST, a receiver operating characteristic (ROC) curve analyses was performed. The effect size (Cohen’s d) and power value (1-β) of the study were calculated using G*Power software. The effect size and power value were 0.72 and 0.99, respectively. A 2-sided* P* value of < 0.05 was considered as significant.

## Results

The mean age of the study population was 55±11 years, and a total of 213 patients were female. The incidence of ST was 3.2% (n*=*42 patients) in the study. The study population was divided into two groups; patients with and without ST (n*=*42 patients vs. n*=*1233 patients, respectively). Table 1 presents baseline demographics characteristics of all patients included in the study. The frequency of hypertension, diabetes mellitus, family of coronary artery disease, and smoking were not different between the groups (*P* > 0.05 for each). However, the frequency of hyperlipidemia was significantly lower in patients with ST (*P* < 0.05). In terms of the usage of previous medications, including acetylsalicylic acid and *B*-blocker, two groups were similar (*P* > 0.05 for each). On admission, patients with ST had lower systolic blood pressure compared to those without ST (*P* < 0.05).

**Table 1:** Baseline characteristics of all patients

	**Total patients, n =1275**	**ST (-), n=1233**	**ST (+), n=42**	***P*** ** value**
Age, year	55 ±11	55±11	59±12	0.008
Female gender, n (%)	213 (16.7)	210 (17)	3 (7.1)	0.091
**Risk factors**				
Hypertension, n (%)	513 (40.2)	495 (40.1)	18 (42.9)	0.725
Diabetes mellitus, n (%)	267 (20.9)	258 (20.9)	9 (21.4)	0.937
Hyperlipidemia, n (%)	532 (41.7)	524 (42.5)	8 (19)	0.002
Family history of CAD, n (%)	288 (22.6)	278 (22.5)	10 (23.8)	0.847
Smoking, n (%)	763 (59.8)	737 (59.8)	26 (61.9)	0.782
**Previous medication**				
Acetylsalicylic acid, n (%)	28 (2.2)	27 (2.1)	1 (2.4)	0.934
*B*-Blocker, n (%)	95 (7.5)	92 (7.5)	3 (7.1)	0.938
ACE inhibitor/ARB, n (%)	255 (20)	247 (20)	8 (19)	0.875
Statin, n (%)	250 (19.6)	245 (19.9)	5 (11.9)	0.201
**On admission**				
Systolic blood pressure, mmHg	133±29	133±29	122±35	0.007
Heart rate, bpm	76±15	76±15	81±14	0.080
Killip class > 1 on admission, n (%)	159 (12.5)	152 (12.3)	7 (16.7)	0.403

Abbreviations: CAD; coronary artery disease, ACE; angiotensinogen converting enzyme, ARB; angiotensinogen receptor blocker. Continuous variables are presented as mean ± standard deviation or median, nominal variables are presented with frequency.

Laboratory and angiographic findings of all patients are shown in Table 2. Patients with ST had a higher levels of peak creatinine kinase myocardial band (*P* < 0.05), while other laboratory findings were similar between the groups (*P* > 0.05 for each). Comparison of interventional outcomes revealed that patients with ST had a higher incidence of left main coronary artery disease (*P* < 0.05). On the other hand, the other angiographic findings were not different between the groups (*P* > 0.05 for each). The median value of the TRI was significantly elevated in patients with ST compared to those without ST (22 [17-32] *vs.* 16 [11-21], *P* < 0.001, respectively). Notably, in-hospital mortality and the length of hospital stay were significantly elevated in patients with ST (*P* < 0.05).

**Table 2:** Laboratory findings and interventional outcomes of all patients

	**Total patients, n =1275**	**ST (-), n=1233**	**ST (+), n=42**	***P*** ** value**
**Laboratory findings**				
WBC count, x10^3^/mm^3^	12.2±3.4	12.2±3.4	12.1±3.1	0.865
Hemoglobin, g/dL	14±1.7	14±1.7	13.9±1.6	0.416
Plasma glucose, mg/dL	143.6±69.7	142.6±67.2	172.1±108.2	0.048
eGFR, mL/min/1.73m^2^	91.4±23.5	91.4±23.5	90.3±22.7	0.370
Peak CK-MB, ng/mL	163 (92-283)	161 (92-278)	216 (123-357)	0.028
Peak troponin I, ng/mL	75 (34-152)	74 (34-151)	99 (48.9-188)	0.087
Left ventricular EF, (%)	47.9±7.8	48±7.7	46.1±8.8	0.116
**Interventional outcomes**				
Door to balloon time, min	31±8	31±8	30±6	0.496
Total ischemia time, min	162 (105-245)	162 (105-245)	147 (83-237)	0.275
LAD as the IRA, n (%)	617 (48.4)	591 (47.9)	26 (61.9)	0.075
Ostial lesion for the IRA, n (%)	659 (51.7)	632 (51.3)	27 (64.3)	0.097
TIMI < 3 flow before pPCI, n (%)	1126 (88.3)	1088 (88.2)	38 (90.5)	0.657
Predilation, n (%)	1012 (79.4)	978 (79.3)	34 (81)	0.797
Stent length, mm	21.1±8.9	21±8.9	22±8.9	0.503
Stent diameter, mm	3.11±0.35	3.11±0.35	3.0±0.33	0.275
Postdilation, n (%)	308 (24.2)	300 (24.3)	8 (19)	0.432
Corrected TIMI frame count	23±13	23±12	27±22	0.869
No-reflow, n (%)	93 (7.3)	89 (7.2)	4 (9.5)	0.572
LMCA disease, n (%)	4 (0.3)	3 (0.2)	1 (2.4)	0.015
Number of diseased vessels, n (%)	1 (1-2)	1 (1-2)	2 (1-2)	0.146
Presence of CTO, n (%)	38 (3)	35 (2.8)	3 (7.1)	0.107
Basal Syntax score	15.7±3.73	15.6±3.72	16.8±3.69	0.061
TIMI risk index	16 (12-22)	16 (11-21)	22 (17-32)	<0.001
In-hospital death, n (%)	71 (5.6)	63 (5.1)	8 (19)	<0.001
Length of hospital stay, day	5 (3-6)	4 (3-6)	5 (4-7)	0.024

Abbreviations: WBC; white blood cell, eGFR; estimated glomerular filtration rate, CK-MB; creatinine kinase myocardial band, EF; ejection fraction, LAD; left anterior descending, IRA; infarct-related artery, TIMI; thrombolysis in myocardial infarction, pPCI; primary percutaneous coronary intervention, LMCA; left main coronary artery, CTO; chronic total occlusion. Continuous variables are presented as mean ± standard deviation or median, nominal variables are presented with frequency.

A multivariate logistic regression analysis was used to determine independent predictors of ST using parameters found to be associated with ST in a univariate analysis (age, female gender, hyperlipidemia, systolic blood pressure, heart rate, plasma glucose, Syntax score, and the TRI). We noted that there was no multicollinearity between the TRI and its continuous parameters (age, heart rate, and systolic blood pressure); hence they were included into multivariate regression analysis together with the TRI. In a multivariate analysis, independent predictors of ST were female gender (Odds ratio [OR]: 0.215, 95% CI: 0.061-0.758; *P* = 0.017), plasma glucose (OR: 1.005, 95% CI: 1.001-1.008; *P* = 0.005) and TRI (OR: 1.061; 95% CI: 1.038-1.085; *P* < 0.001), and these variables were shown in Table 3.

**Table 3:** Multivariate logistic regression analysis of independent predictors of stent thrombosis^a^

	**Odds ratio (OR)**	**95% CI**	***P*** ** value**
Female gender	0.215	0.061-0.758	0.017
Plasma glucose	1.005	1.001-1.008	0.005
TIMI risk index	1.061	1.038-1.085	<0.001

Abbreviations: TIMI; thrombolysis in myocardial infarction. ^a^All relevant variables were included in a multivariate analysis.

**Figure 2:**
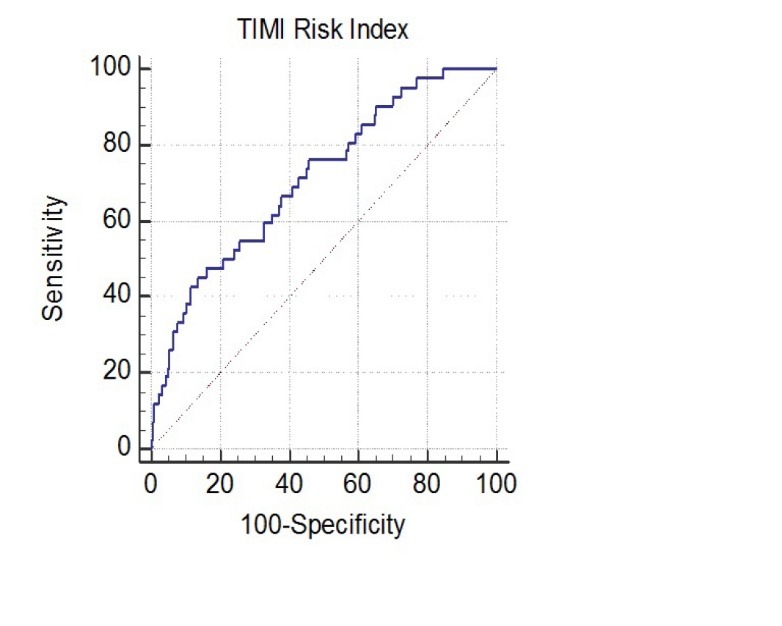
A receiver operating characteristic curve analysis of TRI for the prediction of stent thrombosis (AUC: 0.712, 95% CI: 0.633-0.790, *P*<0.001).

**Figure 3:**
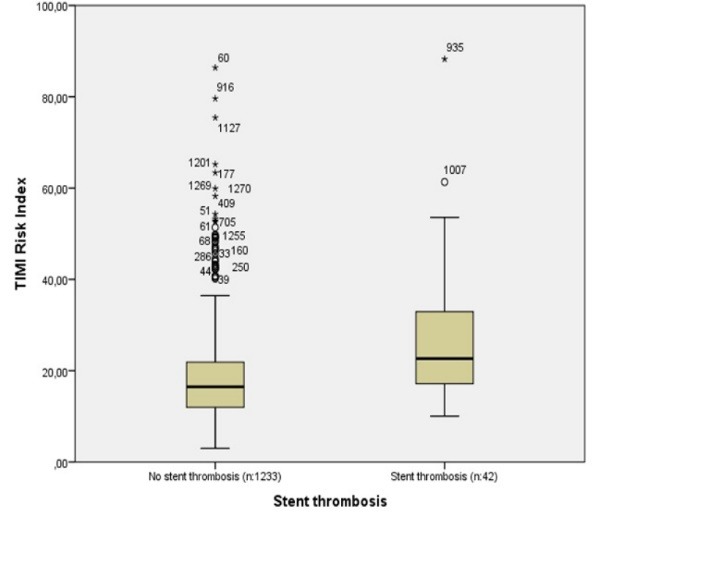
A box plot showing the difference between the TRI values of patients with and without stent thrombosis.

In a ROC curve analysis, the area under the curve (AUC) of TRI was 0.712 (*P* < 0.001, 95% CI: 0.633-0.790). The optimal value of TRI for ST was found to be 25.8 with a sensitivity of 45.2% and a specificity of 86.4% (Figure 2). A box plot was drawn to show the difference between the TRI values of patients with and without ST (Figure 3).

## Discussion

The present study finding has demonstrated that the TRI may be an independent predictor of ST in STEMI patients who were treated with pPCI. To the best of our knowledge, this is the first study in the literature in which the TRI and its relationship with ST was evaluated in STEMI patients treated with pPCI.

ST is an uncommon but life-threating complication of PCI. Particularly, patients who present with ST after STEMI have larger infarction, thereby demonstrating higher mortality rates.^15^ In a previous study, it was reported that the incidence of ST during elective procedures is approximately 1%.^15^ However, the incidence of this fearful complication is much higher in patients presented with acute coronary syndrome, and it may range from 1.3% to as high as % 3.6.^15^ Allying with these prior studies, the incidence of ST was 3.2% in our study.

Some experimental studies have found that a procoagulant state exists in older patients because of the activation of the intrinsic coagulation system, decreased fibrinolytic activity, or alterations in platelet function.^16,17^ Also, it is well-known that older patients with coronary artery disease tend to have more diffused, tortuous, and calcified lesions, which may undoubtedly affect the optimal apposition of the stent to the artery wall, thereby increase the risk of ST.^18^ In common with these study’s findings, a large observational cohort study showed that older patients have elevated risk of ST after pPCI.^19^

Although there have been great improvements in PCI techniques and stent technology in the last two decades, patient’s hemodynamic status upon admission may be related with the elevated mortality risk as well as major adverse cardiovascular events.^20,21^ A recent study has reported that patients with lower systolic blood pressure have higher mortality rates and adverse cardiovascular outcomes – such as ST, recurrent myocardial infarction etc – compared to those with normal systolic blood pressure.^22^ In this study, the researcher hypothesized that the decreases in blood pressure ultimately affect the coronary blood flow, thus causing a higher incidence of ST. Moreover, a previous study showed that hemodynamic instability, namely cardiogenic shock, is an independent predictor of ST after pPCI.^23^

The TRI is a simple bedside score that includes readily available clinical variables such as age, systolic blood pressure, and heart rate. The TRI is mainly designed to be used at initial presentation to predict risk of short as well as long-term mortality in patients with STEMI.^9-11^ Moreover, a recent study showed that STEMI patients with a high TRI have elevated risk for congestive heart failure after index hospitalization.^24^ In a study conducted by Ilkhanoff et al also reported that the TRI may be used to predict short- and long-term mortality in patients with acute coronary syndrome.^25^ On the other hand, it has been unknown whether the TRI can be used to determine the risk of ST in STEMI patients. In the present study, we found a significant difference in ST when comparing patients with a high TRI and those with a low TRI.

Our study findings may point up to significant findings because the TRI is composed of clinical variables that may be used at initial triage in the emergency room without any need for medical history and laboratory analysis. In a clinical practice, patients with a high TRI should be closely followed-up, and more intensive therapy should be given to these patients because of the increase risk of ST. Intravenous, direct- acting P_2_Y_12_ inhibitor may be considered in the acute phase in patients with a high TRI to achieve the rapid deactivation of the platelets. Moreover, this study finding highlights the need whether the TRI has the same relation with ST if ticagrelor is used in patients treated with pPCI since there has been a shift towards using ticagrelor instead of clopidogrel in the PLATO (The Study of Platelet Inhibition and Patient Outcomes) trial.^26^ However, because our study had a retrospective design, multi-center and prospective studies with large population are necessary to clarify the exact role of TRI for ST in STEMI patients treated with pPCI.

### Study limitations

This study has some limitations. First, the study design was retrospective and observational. Second, we only included patients with STEMI; therefore, our study findings may not be generalized to all acute coronary syndrome patients. Third, systolic blood pressure, the component of TRI, was obtained by a non-invasive method. Fourth, although we performed a multivariate logistic regression analysis to determine the independent predictors of ST, some unmeasured confounders may be still present. Fifth, optical coherence tomography or intravascular ultrasonography was not used in all patients for the assessment of the outcome of stenting after pPCI.

## Conclusion

In summary, we tested the predictive value of TRI for the assessment of ST in STEMI patients who underwent pPCI. Based on our results, the TRI might be an independent predictor of ST among these patients. Our findings are important and valuable, because this simple bedside risk index may be used by clinicians to determine the risk of ST in STEMI patients who underwent pPCI.

## Competing interests

None.

## Ethical approval

The study protocol was approved form the Local Ethic Committee (ethic code number: 80576354-050-99/56), and it was performed in accordance with the principles of the Declaration of Helsinki.
